# Determining factors for COVID-19 vaccine hesitancy among Brazilians: a study using structural equation modeling

**DOI:** 10.1590/0034-7167-2024-0112

**Published:** 2024-08-30

**Authors:** Emerson Lucas Silva Camargo, Álvaro Francisco Lopes de Sousa, Anderson Sousa dos Reis, Mariana dos Reis Fortunato, Isaias dos Santos Gouveia, Isabel Amelia Costa Mendes, Carla Aparecida Arena Ventura

**Affiliations:** I Universidade de São Paulo. Ribeirão Preto, São Paulo, Brazil; II Instituto Sírio-Libanês de Ensino e Pesquisa. São Paulo, São Paulo, Brazil; III University Lisbon, Public Health Research Center, Comprehensive Health Research Center. Cidade Universitária, Alameda da Universidade Lisbon, Potugal; IV Universidade Federal da Bahia. Salvador, Bahia, Brazil; V Universidade Federal do Triangulo Mineiro. Uberaba, Minas Gerais, Brazil; VI Universidade de Ribeirão Preto. Ribeirão Preto, São Paulo, Brazil

**Keywords:** Health Behavior, Communication, COVID-19, Disinformation, Global Health, Comportamentos Relacionados com a Saúde, Comunicação, COVID-19, Desinformação, Saúde Global, Conductas Relacionadas con la Salud, Comunicación, COVID-19, Desinformación, Salud Global

## Abstract

**Objectives:**

to investigate the factors influencing vaccine hesitancy against COVID-19 among Brazilians.

**Methods:**

this research employed an observational and analytical approach, utilizing a web-based survey. Data collection took place in 2020, and data analysis was conducted using structural equation modeling.

**Results:**

the prevalence of vaccine hesitancy was found to be 27.5% (1182 individuals). There is a negative correlation between belief in conspiracy theories and social influence. Among the various beliefs associated with vaccination intentions, only conspiracy beliefs exhibited significant predictive value. Thus, the findings suggest that personal beliefs significantly impact hesitancy towards vaccination, and also indicate that trust in governmental bodies is inversely related to hesitancy.

**Conclusions:**

vaccine hesitancy emerges as a multifaceted phenomenon influenced by a complex array of factors, including personal beliefs, trust in governmental bodies, and healthcare systems.

## RESUMO

**Objetivos:**

investigar os fatores que influenciam a hesitação em relação à vacina contra a COVID-19 entre brasileiros.

**Métodos:**

esta pesquisa empregou uma abordagem observacional e analítica, utilizando uma pesquisa online. A coleta de dados ocorreu em 2020 e a análise dos dados foi realizada utilizando modelagem de equações estruturais.

**Resultados:**

a prevalência da hesitação vacinal foi de 27,5% (1182 indivíduos). Há uma correlação negativa entre a crença em teorias da conspiração e a influência social. Entre as diversas crenças associadas às intenções de vacinação, apenas as crenças em conspirações exibiram valor preditivo significativo. Assim, os resultados sugerem que as crenças pessoais impactam significativamente a hesitação em relação à vacinação e também indicam que a confiança em órgãos governamentais está inversamente relacionada à hesitação.

**Conclusões:**

a HV emerge como um fenômeno multifacetado, influenciado por uma complexa gama de fatores, incluindo crenças pessoais, confiança em órgãos governamentais e sistemas de saúde.

## RESUMEN

**Objetivos:**

investigar los factores que influyen en la vacilación ante la vacuna contra la COVID-19 entre los brasileños.

**Métodos:**

esta investigación empleó un enfoque observacional y analítico, utilizando una encuesta en línea. La recolección de datos se llevó a cabo en 2020 y el análisis de los datos se realizó utilizando el modelado de ecuaciones estructurales.

**Resultados:**

la prevalencia de la vacilación ante la vacuna fue del 27,5% (1182 individuos). Existe una correlación negativa entre la creencia en teorías de conspiración y la influencia social. Entre las diversas creencias asociadas con las intenciones de vacunación, solo las creencias en conspiraciones mostraron un valor predictivo significativo. Así, los resultados sugieren que las creencias personales impactan significativamente la vacilación ante la vacunación y también indican que la confianza en los órganos gubernamentales está inversamente relacionada con la vacilación.

**Conclusiones:**

la vacilación ante la vacuna emerge como un fenómeno multifacético influenciado por una amplia gama de factores, incluyendo las creencias personales, la confianza en los órganos gubernamentales y los sistemas de salud.

## INTRODUCTION

Before the official launch of the COVID-19 vaccine, Brazil encountered a complex dilemma regarding vaccine hesitancy (VH), a challenge faced by many other countries as well. Although the first cases in the country were reported in March 2020, it was not until February 2021 that a Brazilian citizen first received the locally manufactured Coronavac vaccine, produced by the Butantan Institute. This study briefly examines the pre-vaccination environment in Brazil^(1-2)^.

The country had to contend with a large number of confirmed cases and deaths, and complicating the situation further, there was also strong resistance to vaccination. By April 2024, Brazil had already reported an impressive 38,757,972 cases and, regrettably, the loss of 711,502 lives due to COVID-19, underscoring the complexity and severity of the issue^(3)^.

The COVID-19 pandemic posed one of the most significant threats to global public health and was notable for its rapid spread and deep social and political ramifications. As a result, there were major disruptions to daily life, an exacerbation of pre-existing psychological issues, and the emergence of new mental health challenges in many nations around the world. In this complex context, achieving optimal vaccine coverage for safety, protection, and disease management was challenging for health systems^(3-8)^.

Previous research has demonstrated how belief systems influence vaccination decisions^(9)^, suggesting a potential mistrust of the disease and its related vaccines. The term “vaccine hesitancy” refers to this doubt, characterized by partial adherence, refusal, or delay in complying with recommended immunization regimes. A complex network of factors, varying in intensity and characteristics over time, including target population, vaccine type, location, epidemiological conditions, misinformation, and adherence to conspiracy theories, contributes to this hesitancy^(8-15)^.

International health authorities continue to confront serious challenges from the dissemination of misleading information about COVID-19 vaccines through conspiracy theories and rumors, as well as from political polarization. The ongoing observation described above complicates the development of herd immunity by influencing VH both directly and indirectly^(11,13-16)^.

Brazil faced a series of challenges that complicated the issue of vaccines. These challenges included an abundance of information, the spread of misinformation, increased scientific skepticism, the endorsement of experimental therapies such as the controversial “chloroquine effect,” political polarization, the propagation of conspiracy theories, and the emergence of an anti-vaccine movement. Together, these factors undermine global efforts to achieve herd immunity and increase vaccination rates, with serious implications for public health^(17-19)^.

In this context, VH emerges as a major global concern, capable of triggering the resurgence of diseases and the onset of new epidemics that result in widespread illness, hospitalizations, and preventable deaths. To fully understand the issues involved, it is imperative to address the underlying reasons for vaccination reluctance. These factors may include gender disparities, educational levels, knowledge of health information, socioeconomic status, racial or ethnic backgrounds, age, generational differences, location, access to technology, information availability, and the efficiency of the health system^(20-23)^.

To effectively combat VH, national and international institutions must collaborate, given the dynamic nature of this disease. This requires a commitment to fulfilling the Sustainable Development Goals (SDGs), outlined in the United Nations framework and referring to social well-being and health. By 2030, this global initiative will serve as a framework for addressing urgent issues, promoting sustainable growth, and raising living standards^(24-26)^.

## OBJECTIVES

To investigate the factors influencing COVID-19 vaccine hesitancy among Brazilians.

## METHOD

### Ethical Considerations

The study was conducted in accordance with national and international ethical standards and received approval from the Research Ethics Committee - CONEP in 2020. The enclosed submission includes the committee’s report. All individuals participating in the online study provided informed consent.

### Research Methodology, Duration, and Geographic Setting

This study is an observational and analytical investigation conducted through an online survey. It involved Brazilian adults residing in the country between May and August 2020. The study design and description presented in this paper were derived from the STrengthening the Reporting of OBservational studies in Epidemiology (STROBE) checklist.

### Inclusion and Exclusion Criteria

The sampling methodology utilized was a modified online version of the snowball method, implemented in two distinct stages: a) Initially, a group of 30 adults was randomly selected from a database of previous studies; b) Subsequently, each of these participants was directed to choose other individuals from their virtual social networks who belonged to the same social category as them^(27-30)^.

To ensure a sample that accurately represents the population, the initial volunteers were selected based on specific criteria to minimize potential biases inherent in population research. These criteria included geographical location (various regions of the country), ethnicity (white and non-white), age group (young, adult, and elderly), and educational level (Elementary/High School, Higher Education, and Post-graduate). Additionally, a strategy for disseminating information was implemented on the social media platform Facebook^®(27-30)^.

The sample size calculation was based on the entire adult population of Brazil, assuming an estimated incidence rate of 50% due to the absence of prior studies on this specific demographic. A margin of error of 3% was applied, along with an adjustment for the sample design effect of 2, and a confidence level of 95%. An additional 20% was included to account for potential losses and refusals. Therefore, the minimum required number of participants was determined to be 2,562 individuals.

### Study Protocol

An online, structured questionnaire, developed by the authors and assessed by experts for cultural and linguistic adequacy, was utilized. The questionnaire, based on scientific literature, encompassed the following blocks for analysis^(25-28)^:

1. Sociodemographic information;
2. Perceptions regarding the COVID-19 pandemic;
3. Information/news seeking and consumption related to COVID-19, including its impact and subsequent actions taken;
4. Agreement with COVID-19 misinformation content;
5. Willingness to receive the COVID-19 vaccine, with reasons for hesitancy if applicable.

The outcome variable (VH), was evaluated through the question: “Will you take the COVID-19 vaccine when it becomes available to the population?” utilizing a dichotomous scale (“yes”/”no”)^(30)^.

### Conceptual Structure and Study Hypotheses:

**Figure 1:**
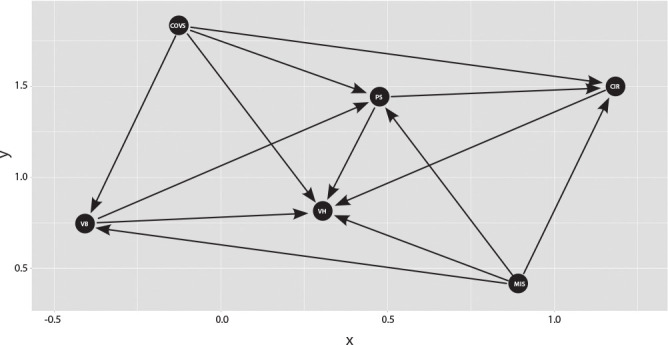
Conceptual Structure of Determinants of COVID-19 Vaccine Hesitancy among Brazilians, Brazil, 2020 COVS - COVID-19 Suffering; MIS - COVID-19 Misinformation; VB - Vaccine Conspiracy Beliefs; PS - Perceived Stress; CIR - COVID-19 Individual Responses; VH - Vaccine Hesitancy.

For the representation of the conceptual structure and hypotheses of the determination of VH for COVID-19, a Directed Acyclic Graphs (DAG) was constructed^(27-30)^ in which latent variables are represented by circles (Figure 1).

Figure 1 presents a conceptual framework outlining factors contributing to VH among Brazilians regarding COVID-19. It includes variables like COVID-19 Suffering (COVS), COVID-19 Misinformation (MIS), Vaccine Conspiracy Beliefs (VB), Perceived Stress (PS), and COVID-19 Individual Responses (CIR), all linked to hesitancy in receiving the COVID-19 vaccine (VH). COVS refers to pandemic-induced suffering, MIS denotes incorrect COVID-19 information, CIR pertains to individual coping strategies, VB represents vaccine-related conspiracy beliefs, and PS indicates stress associated with COVID-19. These factors collectively contribute to VH, with MIS and VB being particularly influential^(30)^.

### Analysis of Results and Statistics

The study employed SPSS software (version 24.0) for preliminary descriptive data analysis and subsequently converted the data to Mplus software (version 8.4) for conducting Structural Equation Modeling (SEM) analyses. Factorial structure was assessed by measuring latent components using Exploratory Factor Analyses (EFA) and Exploratory Structural Equation Models (ESEM). Confirmatory Factor Analysis (CFA) was then performed to validate the observed dimensionalities.

The criteria for accepting an item comprised factor loadings of 0.3 or higher and residual variances of 0.7 or below, both of which were standardized. The evaluation was conducted on the structural model, which consists of latent variables, measurements, and directly observed variables. Regression coefficients, both raw and normalized, were estimated using a significance threshold of 5%. The effects were classified as weak/small (about 0.10), moderate/medium (around 0.30), and strong/large (beyond 0.50).

The analyses were performed on both the general population and particular subgroups, including gender, age group, and educational level. The Weighted Least Squares Means and Variance Adjusted (WLSMV) estimator was used for these analyses. The model definition involved assessing Modification Indices (MI≥10) and Expected Parameter Changes (EPC≥0.25). The fit indices used for model evaluation included the Root Mean Square Error of Approximation (RMSEA), which should be less than 0.06 (ideally less than 0.08 with a 90% confidence interval below 0.08), the Comparative Fit Index (CFI), which should be greater than or equal to 0.95, and the Tucker-Lewis Index (TLI), which should also be greater than or equal to 0.95.

## RESULTS

The social and demographic characteristics of the participants are presented in Table 1. VH was observed in 27.5% of the population, specifically 1182 individuals. The findings indicate a favorable inclination towards vaccine acceptance among the participants under study, however a notable proportion still exhibits hesitancy or outright refusal to undergo vaccination.

**Table 1:** Social and Demographic Characteristics of Brazilians During the COVID-19 Pandemic, Brazil, 2020

Variable	n	%
Age Range		
18 to 29 years old	1577	36.8
30 to 49 years old	1947	45.4
50 or older	767	17.9
Marital Status		
In a relationship	2766	64.5
Single	1525	35.5
Gender		
Male	1056	24.6
Female	3209	74.8
Non-binary	18	0.4
I don’t use a specific term	8	0.2
Education		
Elementary and High School	804	18.8
College	1295	30.3
Postgraduate, Master’s or Doctorate	2172	50.9
Religion		
No	1339	31.2
Yes	2952	68.8
Agreement with the need for social distancing/quarantine		
Completely disagree	12	0.3
Disagree	56	1.3
Neither agree nor disagree	64	1.5
Agree	914	21.3
Completely agree	3245	75.6
Agreement with the strategies adopted by your local government to face the pandemic?		
Completely disagree	385	9
Disagree	1079	25.1
Neither agree nor disagree	236	5.5
Agree	1810	42.2
Completely agree	781	18.2
Fear of the consequences of the pandemic		
No	326	7.7
Yes	3917	92.3
Are you in social isolation due to COVID-19?		
No	386	9
Partial isolation	1577	36.8
Total isolation	2328	54.3
How long have you been in social isolation?		
I’m not in isolation	417	9.7
60 days or less	949	22.1
Between 61 and 90 days	578	13.5
More than 90 days	2347	54.7
Do you believe that COVID-19 has an impact on your life?		
Little impact	418	9.7
Medium impact	1445	33.7
High impact	2348	54.7
I can’t answer	80	1.9

Table 2 displays the magnitude and orientation of connections between hidden and observable factors. For instance, when observable variables have larger coefficients for COVID-19 Individual Responses (CIR), it indicates that there are greater beliefs or actions made in response to COVID-19. The p-values and confidence ranges illustrate the statistical significance of these connections.

**Table 2:** Standardized direct and indirect coefficients for variables associated with COVID-19 vaccine hesitancy among Brazilians, 2020

Lhs	op	Rhs	est.std	Se	z	*p* value	ci.lower	ci.upper
CIR	=~	Bars	0,42	0,01	22,60	0,00	0,38	0,45
CIR	=~	Disinfectants	0,39	0,01	20,97	0,00	0,36	0,43
CIR	=~	Hygiene	0,26	0,02	12,20	0,00	0,22	0,30
CIR	=~	Travel	0,51	0,01	29,96	0,00	0,47	0,54
CIR	=~	Remote work	0,46	0,01	28,02	0,00	0,43	0,50
CIR	=~	Supply	0,37	0,01	26,79	0,00	0,35	0,40
CIR	=~	Social isolation	0,47	0,01	25,07	0,00	0,43	0,51
CIR	=~	Masks	0,07	0,02	2,70	0,00	0,020	0,12
CB	=~	Chinese	0,73	0,00	80,23	0,00	0,71	0,75
CB	=~	Reduced isolation	0,41	0,01	27,36	0,00	0,38	0,45
CB	=~	Asymptomatic	0,32	0,01	19,301	0,00	0,29	0,35
CB	=~	Pharmaceutical	0,85	0,00	112,00	0,00	0,84	0,87
GB	=~	Hibiscus tea	0,74	0,00	86,23	0,00	0,72	0,76
GB	=~	Alcohol	0,40	0,01	25,87	0,00	0,37	0,43
GB	=~	Toxic alcohol	0,54	0,01	42,06	0,00	0,51	0,56
GB	=~	Ingestion	0,82	0,00	114,82	0,00	0,81	0,84
GB	=~	Vinegar	0,77	0,00	86,90	0,00	0,75	0,78
GB	=~	Autohemotherapy	0,56	0,01	47,39	0,00	0,54	0,59
GB	=~	Eating garlic	0,82	0,00	110,35	0,00	0,80	0,83
GB	=~	High temperature	0,63	0,01	57,50	0,00	0,61	0,66
GB	=~	Drinking water	0,81	0,00	128,07	0,00	0,80	0,83

*lhs - Left Hand Side; op - Operator; rhs - Right Hand Side; est.std - Standardized Estimate; se - Standard Error; z - Z-statistic; ci.lower - Lower Confidence Interval; ci.upper - Upper Confidence Interval; CIR - COVID-19 Individual Responses; CB - Conspiracy Beliefs; GB - Government Beliefs.*

**Figure 2:**
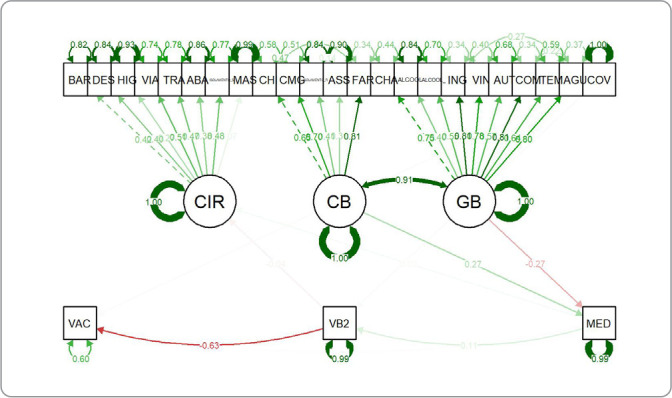
Models with direct and indirect associations between the tested variables and vaccine hesitancy for COVID-19 among Brazilians, 2020 CIR - COVID-19 Individual Responses; CB - Conspiracy Beliefs; GB - Governmental Beliefs; VB2 - Vaccine Conspiracy Beliefs; VAC - VACCINATION (Intends to voluntarily vaccinate against COVID-19); MED - FEAR.

Figure 2 depicts a structural equation model for VH. The main findings indicated by the figure are the various standardized path coefficients between observed variables (such as behaviors and beliefs regarding COVID-19) and latent constructs (CIR, CB, and GB). The numbers on the paths represent the strength of the association between the variables, and the direction of the arrows shows the direction of hypothetical influence. Red lines typically indicate negative associations, while green lines indicate positive associations. The model also includes the vaccination variable (VAC), showing its relationship with these constructs. This figure is a visual representation of the complex relationships and influences on VH among the studied population.

During our analysis of VH among Brazilians using structural equation modeling, we found that out of the several beliefs related to vaccination intentions (VAC), only Conspiracy Beliefs (VB2) had a significant predictive value. COVID-19 Individual Responses (CIR) were not statistically significant, except when they were linked to VB2. Fear (MEDO) was found to be a significant influence within the scope of VB2. The model clarifies the complex nature of vaccine hesitation, emphasizing an inverse relationship between conspiracy beliefs (CB) and social influence (CIR). The findings indicate a complex relationship, where faith in political organizations (GB) is negatively correlated with hesitancy, whereas trust in healthcare systems (CB) is positively associated. The standardized loadings highlight the fact that personal views have a major impact on the hesitancy to vaccinate, thereby stressing the intricate nature of the various factors that influence public health behaviors.

## DISCUSSION

The objective of this study was to examine the factors contributing to Brazilian hesitancy toward accepting a COVID-19 vaccine. The results indicated that, before the vaccination campaign in Brazil, at least one-third of participants exhibited hesitancy toward COVID-19 vaccines. The determining factors showed that VH is a complex issue influenced by multiple factors, including differences in knowledge, perceived risk, trust in medical authorities, experiences during the epidemic, and social influences^(31-33)^.

The rate of COVID-19 VH among Brazilians aligned with similar percentages reported in the literature, which varied from 4.3% to 72% across different groups^(27-33)^. However, the result in our sample was considerably higher than the prevalence found in other Portuguese-speaking nations (21.1%)^(30)^. The increased frequency in Brazil could be attributed to factors unique to the country, including trust in authorities within a politically divided context, the spread of misinformation and misconceptions about the effectiveness and safety of vaccines, and cultural and religious convictions related to vaccination^(34-39)^.

Several studies have aimed to understand the factors contributing to VH in specific communities. Biswas et al.^(34)^ conducted research on VH among healthcare professionals and determined that the frequency of hesitancy was 22.5%. In contrast, Veronese et al.^(40)^ conducted a study with an older group and found that hesitancy levels were similar to ours (27.3%). This hesitancy was mainly due to concerns about vaccine efficacy and adverse effects. Based on a comprehensive analysis, Fajar et al.^(41)^ determined that the global prevalence of the phenomenon in the general population is 25%. In general, this prevalence can be related to various factors, such as cultural beliefs, government regulations, trust in medical professionals, and the impact of the pandemic in different countries.

Our study highlights interesting relationships between latent variables. For example, there is a negative association between belief in conspiracy theories (CB) and social influence (CIR). This suggests that individuals who subscribe to conspiracy theories may be less swayed by social factors, whether positive or negative. A plausible explanation is that these beliefs are often linked to a profound disdain for dominant narratives and a general reluctance to adhere to social norms. Additionally, conspiracy theories can offer an alternative viewpoint and a sense of control over complex events, such as a health crisis. Consequently, individuals who embrace these beliefs may distance themselves from social standards and health guidelines, such as vaccination^(32,38,42)^.

On the other hand, we discovered that trust in governmental bodies (GB) is crucial for vaccine acceptance. Our model shows a negative correlation between VH and this variable, indicating that as people’s trust in the government increases, their hesitation or reluctance to receive vaccines decreases. This is consistent with findings in the literature^(43-44)^, which demonstrate that the perception of governments as capable and trustworthy in disseminating accurate information can lead to greater acceptance of public health programs. This is particularly relevant in situations where the state supports immunization programs and the government endorses or mandates public health actions^(43,45)^.

The relationship between VH and trust in the healthcare system is clear but may appear paradoxical. Normally, one would expect that greater trust in healthcare systems would lead to higher vaccine acceptance. However, this observation suggests that people may not perceive the need for preventive vaccines because they believe the healthcare system can effectively manage the disease^(46-48)^. This may also indicate that while people trust healthcare systems to meet their needs, they may harbor reservations about the rapid development of COVID-19 vaccines and whether the systems are approving them without adequately considering the risks or long-term consequences^(46,49-50)^.

Overall, our model indicates that VH is a complex and multifaceted issue influenced by social, personal, psychosocial, and socio-political factors. To manage VH effectively, it is necessary to implement a comprehensive and targeted approach that addresses not only vaccine safety and efficacy but also strengthens public trust in health institutions and governments while combating misinformation. The dissemination of accurate and favorable information about vaccines can be promoted through enhanced communication techniques, public awareness initiatives, and the involvement of community leaders and influential individuals^(23,36)^.

This study aligns with and directly contributes to Sustainable Development Goal 3 (SDG 3), which aims to ensure healthy lives and promote well-being for all. By analyzing factors that impact VH among Brazilians, particularly in relation to COVID-19, our findings aid in preparing for future health crises and improving vaccination rates. One of the main objectives of SDG 3 is to improve global public health and reduce mortality rates associated with pandemics^(24)^. Additionally, this study aligns with SDG 16, which supports inclusive and peaceful societies for sustainable development. It elucidates how misinformation and trust in health institutions impact vaccine acceptance and suggests actions to strengthen public trust in these institutions.

### Study limitations

We recognize that our work has limitations. First, it is subject to selection bias since only those with internet access and digital literacy could accept and participate in the study. Additionally, the observational nature of the study and the approach of collecting data through an online survey may limit the sample’s representativeness and its generalizability.

It is also important to highlight the limitations of the snowball sampling method. Although it is an effective strategy for reaching a diverse sample, it can result in the homogenization of responses. The self-reporting of responses can also lead to social desirability bias, where individuals may respond in a manner they believe to be more socially acceptable. However, this can be minimized by the online collection method. Finally, given the complexity of the factors influencing VH and the interaction between these factors, it is crucial to interpret the results with caution. These results represent a snapshot of a specific period (before the start of vaccination) and may not accurately reflect the dynamics of constantly changing public attitudes toward vaccination.

### Contributions to the Nursing Field

Our research makes a significant contribution to the science and practice of nursing, especially in areas related to crisis management and public health, such as the COVID-19 pandemic. By identifying factors that cause Brazilians to hesitate to receive COVID-19 vaccines, we highlight the critical role nurses play in promoting the health of individuals, families, and communities. Understanding the complex interactions of variables such as misinformation, conspiracy theories, perceived stress, and personal reactions to the epidemic, nurses can develop more effective and culturally aware communication techniques. These tactics are aimed at debunking myths about vaccines and increasing public trust in health interventions. Our findings also provide support for nurses to advocate for robust health policies and tailored educational programs that address specific issues and needs, thereby promoting a more coordinated and successful response to public health emergencies.

## CONCLUSIONS

Our research indicated a high prevalence of HV for COVID-19 among Brazilians before the start of the vaccination period. Our findings suggest that VH is a multifaceted issue influenced by a range of psychological variables, personal views, and belief systems. Interventions must be specific, culturally sensitive, focused, and diversified to effectively address HV. They should address concerns about the efficacy and safety of vaccines, as well as build public trust in medical and governmental authorities. Moreover, to dispel misinformation and conspiracy theories, the use of persuasive communication techniques, public education initiatives, and the involvement of social influencers and community leaders is necessary.
